# Chinese herb mix Tiáo-Gēng-Tāng possesses antiaging and antioxidative effects and upregulates expression of estrogen receptors alpha and beta in ovariectomized rats

**DOI:** 10.1186/1472-6882-11-137

**Published:** 2011-12-30

**Authors:** Lian-wei Xu, Lan Kluwe, Ting-ting Zhang, Sheng-nan Li, Yan-yan Mou, Zhen Sang, Jun Ma, Xiong Lu, Zhuo-jun Sun

**Affiliations:** 1 Gynecology Department, Shanghai Yueyang Integrated Traditioanl Chinese Medicine and Western Medicine Hospital affiliated to Shanghai University of Traditional Chinese Medicine, Gan He Road 110, Shanghai, China; 2 HanseMerkur Traditional Chinese Medicine Centre at University medical centre Hamburg-Eppendorf, Martinistrasse 52, Hamburg, Germany; 3 Laboratory for Research and Diagnostics, Department of Maxillofacial Surgery and Neurology, University Medical Center Hamburg-Eppendorf, Martinistrasse 52, Hamburg, Germany; 4 Gynecology Department, Shanghai Shuguang Hospital affiliated to Shanghai University of Traditional Chinese Medicine, Pu An Road 185, Shanghai, China; 5 Shanghai University of Traditional Chinese Medicine, Cai Lun Road 1200, Shanghai, China

## Abstract

**Background:**

Herb mixtures are widely used as an alternative to hormonal therapy in China for treatment of the menopausal syndrome. However, composition of these herb mixtures are complex and their working mechanism is often unknown. This study investigated the effect of Tiáo-Gēng-Tāng (TG-decoction), a Chinese herbal mixture extract, in balancing female hormones, regulating expression of estrogen receptors (ERs), and preventing aging-related tissue damage.

**Methods:**

Ovariectomized 5-month-old female rats were used to model menopause and treated with either TG-decoction or conjugated estrogen for 8 weeks. Estradiol (E_2_), luteinizing hormone (LH) and follicle-stimulating hormone (FSH) were measured in serum and in the hypothalamus. Hypothalamic expression of estrogen receptor (ER) alpha and beta were studied by real-time PCR and western blotting. Total antioxidant capacity (T-AOC), oxidation indicator superoxide dismutase (SOD) activity and tissue damage parameter malondialdehyde (MDA) were measured using standard assays. Aging-related ultrastructural alterations in mitochondria were studied in all animals by transmission electron microscopy.

**Results:**

TG-decoction-treatment elevated E_2 _and lowered FSH in serum of ovariectomized rats. The potency and efficacy of TG-decoction on the hypothalamus was generally weaker than that of conjugated estrogens. However, TG-decoction was superior in upregulating expression of ERα and β. TG-decoction increased hypothalamic SOD and T-AOC levels and decreased MDAlevels and mitochondrial damage in hypothalamic neurons.

**Conclusions:**

TG-decoction balances female hormones similarly to conjugated estrogens but less effectively. However, it is superior in up regulating ERα and β and exhibits antioxidative antiaging activities. Whilst it shares similar effects with estrogen, TG-decoction also seems to have distinctive and more complex functions and activities.

## Background

Estrogen therapy is regarded as standard treatment for the menopausal syndrome, which is caused by rapid decrease and fluctuation of female hormones [1]. However, estrogen substitution is also associated with side effects including endometrial hyperplasia, increased risk of certain cancer types (breast and ovarian cancer and endometrial carcinoma), liver abnormalities, and hematological adverse effects (coronary heart disease, stroke and venous thromboembolism) [2-7]. Recently, alternative therapies using natural products with milder efficacy but fewer side effects have been studied, herb mixtures of traditional Chinese medicine (TCM) being one such alternative therapy studies.

Tiáo Gēng Tāng (TG-decoction) meaning "decoction which regulates body balances in menopause", is among these Chinese herb mixtures, and has been used to treat symptoms of menopause in China for more than 30 years. According to the TCM theory, the menopausal syndrome is caused by kidney-liver weakness-based yin-yang imbalance and organ disharmony [8]. TG-decoction consists of 10 herbs (Table 1) and is composed to strengthen kidney and liver function, to nourish deficient essence in organs, to disperse excessive fire from the body, and finally to balance yin and yang.

**Table 1:** Compositions of TG-decoction

Chinese name	Ping Yin	Latin name	Part used	daily adult Dose (g)
生地黄	*shēng dì huáng*	*Radix rehmanniae*	Dried root	15
白芍	*bái sháo*	*Radix paeoniae alba*	Dried root	10
巴戟天	*bā jí tiān*	*Radix morindae officinalis*	Dried root	9
淫羊藿	*yín yáng huò*	*Herba epimedium*	Dried herb	15
知母	*zhīmǔ*	*Rhizoma anemarrhenae*	Dried rhizome	9
黄柏	*huáng bǎi*	*Cortex phellodendri*	Dried bark	9
生龙骨	*shēng lóng gǔ*	*Os draconis*	Fossil bone	30
生牡蛎	*shēng mǔ lì*	*Concha ostreae*	shell	30
夜交藤	*yè jiāo téng*	*Caulis polygoni multiflori*	Dried vine	30
柴胡	*chái hú*	*Radix bupleurum*	Dried root	9
			Total amount:	166

In conventional medicine, this alternative rationale is difficult to be explained scientifically although conventional medicine and TCM share a common understanding of pathological mechanisms of menopause to be an aging-related disorder with decreasing estradiol (E_2_) levels. E_2 _contributes to activate the antioxidant defense systems, scavenging reactive oxygen species (ROS) and thereby reduces aging-related damage in mitochondria, a key organelle in the development of aging-associated cellular damage [9,10]. Oxidative stress in the process of aging particularly affects the brain due to its high metabolic rate and reduced capacity for restoration compared to other organs [11].

Empirically and as studies have proven, TG-decoction is especially effective for treatment of hot flashes, sleep disturbance and emotional instability during menopause, with success rates of 66-90% [12]. It is known that TG-decoction elevates E_2 _and lowers follicle-stimulating hormone (FSH) in postmenopausal women [12]. These effects are similar to those of estrogen therapy and may explain some effects of the drug. However, more detailed studies on TG-decoction and its effects and side effects are lacking.

TG-decoction provides systemic therapy, and antiaging and antioxidative effects are expected and are addressed in the present study. We used ovariectomized rats as a model for the menopause and focused on the hypothalamus because this is the regulatory center for the neuroendocrine system [13], thermoregulation [14,15], circadian rhythms [16], emotion [17], autonomic nervous system [18], and other functions that are frequently affected by menopausal hormonal changes [19]. We further studied estrogen receptor (ER) α and β because previous works have hypothesized that decreased expression of these receptors contribute to reduced responsiveness of hypothalamus to estradiol during aging [20,21].

## Methods

### Preparation of TG-Decoction

TG-decoction is a decoction of 10 Chinese herbs as listed in Table 1. A typical daily dose for an adult is 166 g. All herbs were purchased from the Pharmacy Department of Shanghai Shuguang Hospital affiliated with Shanghai University of Traditional Chinese Medicine, China. The decoction was prepared in the laboratory of the Science and Technology Experiment Centre of Shanghai University of Traditional Chinese Medicine. All herbs were soaked in water in a ratio of 1/3 (g/ml) for 30 min and subsequently boiled for a further 30 min. After saving the supernatant containing constituent extracted from the herbs, the same volume of water was added to the dregs and boiled for a further 30 min. The two extracts were pooled together and concentrated by means of heating evaporation. The volume of the decoction was adjusted to 2 ml for each rat. The decoction was stored at 4°C until the experiment.

### Animals and treatment

All experimental procedures were evaluated and granted ethical approval by the National Natural Science Foundation, Beijing, China (approval number: 30772820). Rats were purchased from the Experimental Animal Centre of Shanghai University of Traditional Chinese Medicine (Certificate of Conformity: SCXK (Shanghai) 2008-0016) and cared for in the specific pathogen free conditions in the center. A total of 72 specific pathogen free Sprague-Dawley female rats were used. Three months after birth at a weight of 200 ± 10 g, 54 rats were bilaterally ovariectomized to create a model of the menopause [22], while the other 18 were left as young non-ovariectomized controls. The 54 ovariectomized rats were randomly divided into 3 groups (18 rats each) and assigned to: (1) untreated controls; (2) TG-decoction treatment; and (3) Premarin (conjugated estrogens tablets; Wyeth Pharmaceutical Co. Ltd., China) treatment. Treatments were started 1 week after bilateral ovariectomy under anesthesia with 3% sodium pentobarbital.

Using 166 g as the daily dose of dried herbs for an adult, the relative body surface-area ratio of rats to humans of 7:1, adult human weight of 60 kg and rat weight of 0.2 kg, the daily dose of dried herbs for one rat was calculated as 3.87 g = 166 g*7*(0.2 kg/60 kg), and that of Premarin as 0.015 mg/rat. TG-decoction was given in 2-ml suspensions (corresponding to 3.87 g dried herbs) intragastrically once daily. Premarin was given the same way. Physiological saline was given to rats in the control group and to rats in the non-ovariectomized group. One day after 8 weeks treatment, serum and hypothalamus were sampled from all the treated and untreated rats for the following measurements. All specimens and samples were stored at -80°C until used for assay.

### Laboratory measurement

FSH, LH and E_2 _in serum and hypothalamus were measured using standard radioimmunoassay and reagents (North Biotechnology Research Institute, Beijing, China) in the laboratory of the Science and Technology Experiment Centre of Shanghai University of Traditional Chinese Medicine. The measurement was carried out according to the protocol of the manufacturer.

SOD was measured using xanthine oxidase method, in which superoxide anion radicals lead to oxidation of hydroxylamine, resulting in a purple nitrite compound measurable by a spectrophotometer. SOD activity leads to reduction of the nitrite compound and thus lowers the absorbance. T-AOC assay was based on reduction of Fe^3+ ^to Fe^2+^, whereas the latter forms complexes with phenanthroline substances, which can be measured using a spectrophotometer. MDA is measured using the thiobarbituric acid method in which MDA, the degradation product of lipid peroxidation, condenses with penthiobarbital and leads to a red product with measurable absorbance using a UV spectrophotometer.

Commercial assay kits for SOD, T-AOC and trace MDA were provided by Jiancheng Biotechnology Research Institute (First Branch) (Nanjing, China). Measurements were performed according the protocol provided by the manufacturer in the laboratory of the Science and Technology Experiment Centre, Shanghai University of Traditional Chinese Medicine.

### Expression of ERα and β

Total RNA was prepared from the hypothalamus using TRIZOL reagent (Promega, Madison, WI, USA) and checked for quality by running them on a 1.2% agarose gel. Reverse transcription was carried out using PrimeScript RT Reagent Kit (TaKaRa BIO, Dalian, China) and subsequent real-time PCR was performed using SYBR Green Supermix (Bio-Rad, Hercules, CA, USA) with an iCycler^® ^Thermal Cycler (Bio-Rad). GADPH was used as an internal reference to normalize levels of total mRNA in a sample. Relative expression of ERα and β in ovariectomized rats (untreated, Premarin-treated or TG-decoction-treated) was calculated against expression of ERα and β in non-ovariectomized rats. Primer sequences are available upon request.

Western blotting was performed as described previously [23]. For western blot analysis, protein was extracted from the hypothalamus, run on denaturing SDS-polyacrylamide gels and transferred onto blotting membranes. After blocking non-specific binding, the membranes were sequentially incubated with primary antibodies for ERα or β (ABCAM, Boston, MA, USA), alkaline phosphatase-labeled secondary antibodies for the primary antibodies and visualized using BCIP/NBT as substrate (Promega). β-Actin was used as a protein quality and quantity control.

### Ultrastructure of hypothalamus mitochondria

Sample preparation was carried as described previously [24]. Immediately after sampling, hypothalamus pellets were placed in ice-cold 2.5% glutaraldehyde solution for 2 h. The specimens were washed four times in 0.2 M cacodylate buffer, and post-fixed in 1% OsO_4 _for 2 h, then dehydrated in graded acetone steps, and embedded in Spurr's resin. Ultrathin sections were double stained with 2% uranyl acetate and lead citrate, and examined by transmission electron microscopy (Tecnai-12 Biotwin; Philips-FEI, Eindhoven, Netherlands) at magnifications of up to 43,000.

### Statistical analysis

Statistical analysis was carried out using SPSS15.0. Data with normal distribution were analyzed using one-way analysis of variance and least significant differences. Data that were not normally distributed were analyzed using the non-parametric Kruskal-Wallis and least significant difference tests. P < 0.05 was considered statistically significant.

## Results

### TG-decoction regulates sexual hormones

**Figure 1:**
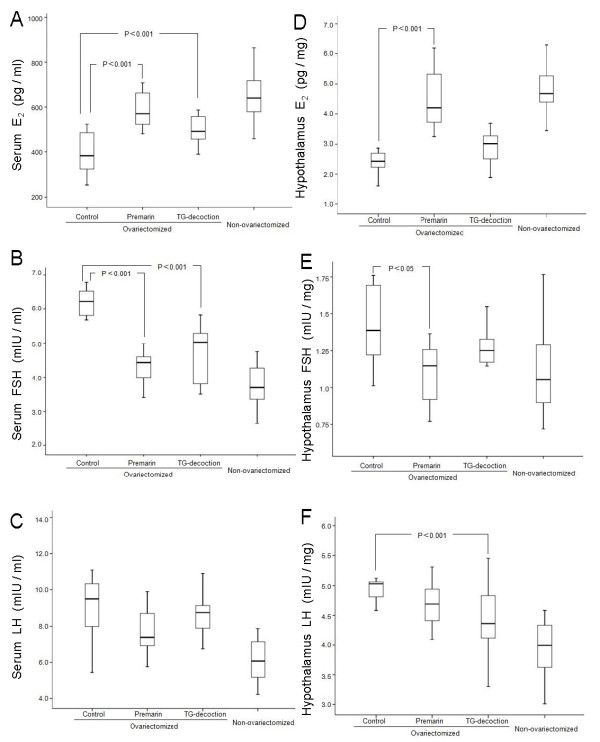
**Sex hormones**. Female hormones E_2_, FSH and LH in serum (A-C) and in the hypothalamus (D-F) in untreated (control), Premarin-treated and TG-decoction-treated ovariectomized rats, and in non-ovariectomized rats. Significance levels of differences are indicated in the graphs.

Bilateral ovariectomy significantly lowered E_2 _levels and elevated FSH and LH in serum and hypothalamus of 5-month-old female rats (Figure 1), providing a menopause model [22].

When these ovariectomized rats were given TG-decoction or Premarin (conjugated estrogens), hormones in serum were regulated towards the normal status of non-ovariectomized control rats, meaning that E_2 _was increased while FSH was decreased (Figure 1A, B). The hormone-regulating effects of TG-decoction were generally weaker than those of Premarin. For example, increased E_2 _and decreased FSH in the hypothalamus were only significant in rats treated with Premarin but not in those treated with TG-decoction (Figure 1D, E).

### Expression of ERα and β

**Figure 2:**
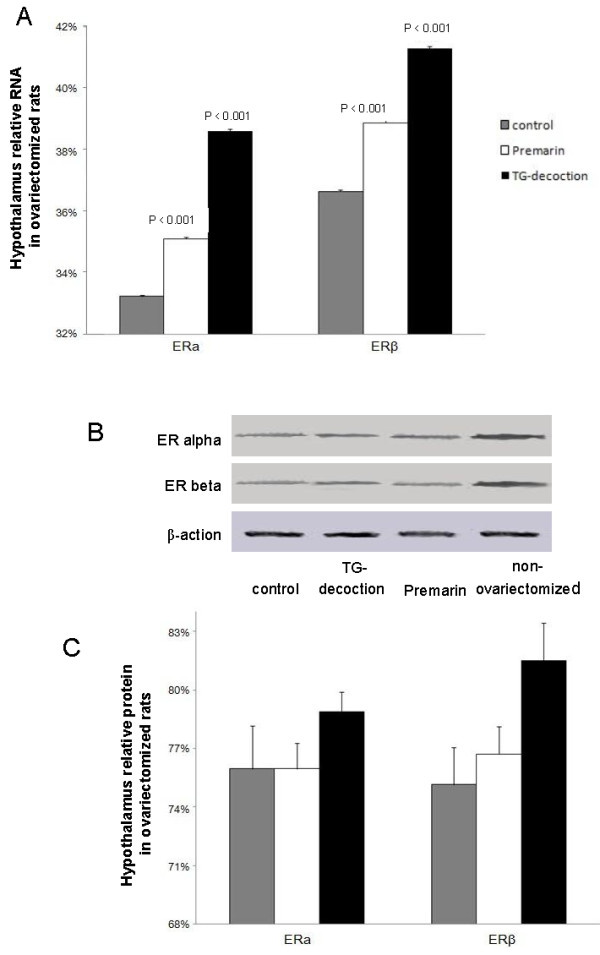
**Expression of ERα and β**. ERα and β in ovariectomized rats at RNA (A) and protein (B, C) levels. B: Western blotting. Gray bars = untreated, white bars = Premarin-treated, black bars = TG-decoction-treated ovariectomized rats. All values are relative to those of non-ovariectomized rats.

Both TG-decoction and Premarin slightly increased ERα and β in ovariectomized rats at the mRNA and protein levels. The effect of TG-decoction was stronger than that of Premarin (Figure 2).

### Antioxidant activity and lipid peroxidation damage

**Figure 3:**
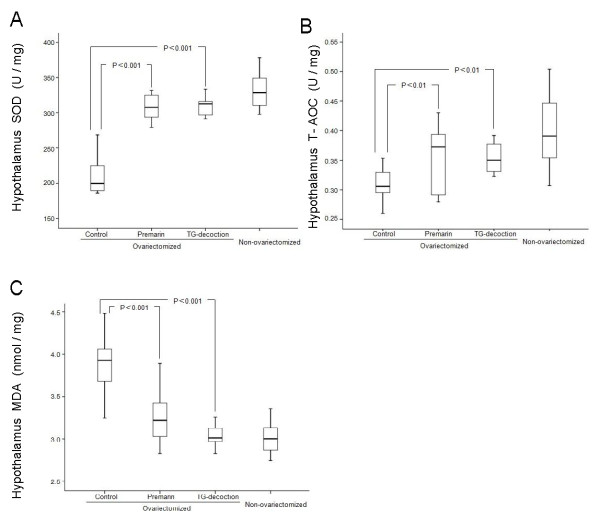
**Antioxidant activity and peroxidation damage**. Antioxidant activity parameters SOD, T-AOC (A, B) and peroxidation damage parameter MDA (C) in untreated (control), Premarin-treated, TGT-treated ovariectomized rats, and in non-ovariectomized rats. Significance levels of differences to the control group are indicated in the graphs.

Bilateral ovariectomy significantly lowered SOD, one of the most important antioxidative enzymes [25] and total antioxidant capacity (T-AOC) [26], and elevated the indicator of lipid peroxidation damage malondialdehyde (MDA) [27]. Both TG-decoction and Premarin significantly reversed these aging parameters by elevating SOD and T-AOC and lowering MDA in the hypothalamus of ovariectomized rats (Figure 3).

### Ultrastructural mitochondria alteration

**Figure 4:**
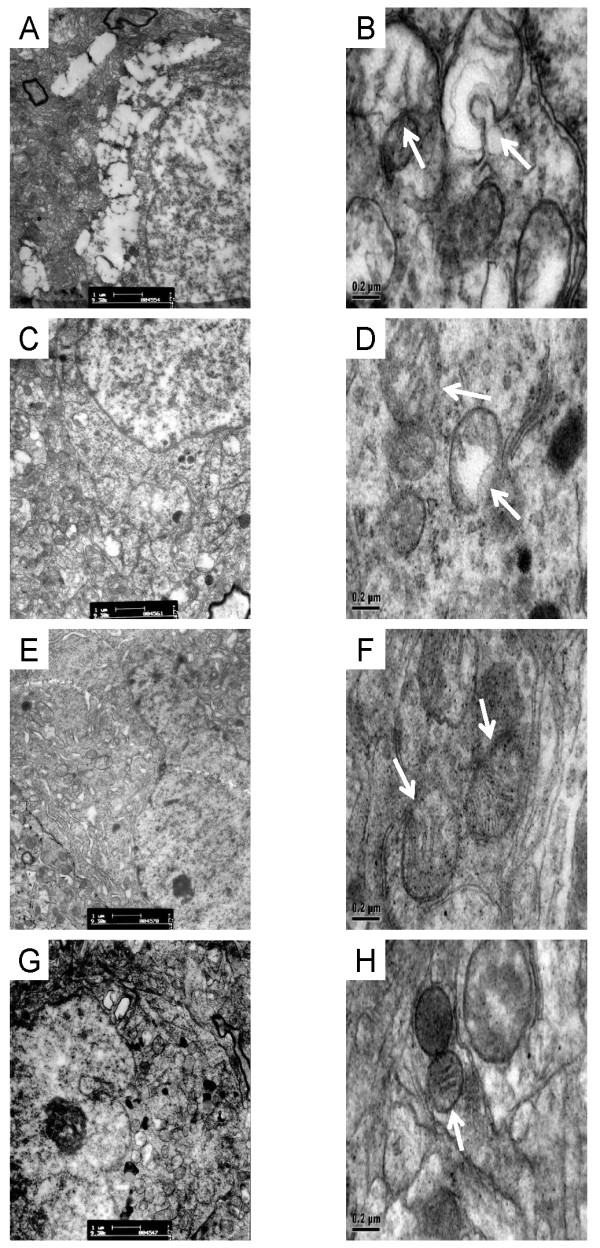
**Ultrastructural mitochondria alteration**. Ultrastructure of mitochondria of hypothalamic neurons in untreated (control), Premarin-treated, and TGT-treated ovariectomized rats, and in non-ovariectomized rats, under electron microscopic magnification of 8,200 (A, C, E, G) and 43,000 (B, D, F, H). Mitochondrial damage is indicated by arrows.

Electron microscopy revealed mitochondrial damage in hypothalamic neurons of ovariectomized rats, characterized by edema, swelling and vacuolization. Also, breakage of cristae was visible (Figure 4B, arrows). Both Premarin and TG-decoction treatment reduced these ultrastructural alterations (Figure 4D, F).

## Discussion

Using ovariectomized rats as a model for the menopause, we found that TG-decoction (1) elevated E_2 _in serum but not in the hypothalamus; (2) had antioxidative activity and tissue-protective function; and (3) upregulated expression of ERα and β in the hypothalamus.

The mild estrogen-like effects of TG-decoction are reasonable because several of its constituents are known to contain flavonoid aglycones (kaempferol, apigenin, quercetin, luteolin and breviflavone B), which have estrogen-like activity [28]. The complete composition of this decoction is being elucidated in ongoing studies that may identify additional compounds with estrogen-like effects.

Results of previous studies regarding expression of ERs in ovariectomized rats and upon E_2 _treatment are controversial [29-32]. In our study, expression of ERs decreased in the hypothalamus of ovariectomized rats and increased upon treatment with TG-decoction. Similar results have been recently reported by Wu *et al*. [33] who have found that total flavonoids of *Epimedium sagittatum *reversed the markedly reduced ERα expression in the hypothalamus of ovariectomized rats. The impact of ovariectomy and estrogen treatment on the expression of ERα and β varies in different parts of the rat brain [34,35]. Also, within the hypothalamus, ER expression may differ in various subregions during aging [20,21]. Further studies are necessary to find out how the expression of ERs in various subregions of the hypothalamus is influenced by the TG-decoction.

TG-decoction exhibited antiaging and tissue-protective activities in this study. Some of these activities may be direct and not mediated by ERs. In fact, antioxidative and cellular protective activities have been reported previously for several constituents of TG-decoction including *yín yáng huò (Herba epimedium) *[36], *bā jí tiān (Radix morindae officinalis) *[37], *shēngdì huáng(Radix rehmanniae) *[38] and *zhīmǔ (Rhizoma anemarrhenae)*[39,40]. Phytoestrogens, for example, genistein in soya, are also known to be antioxidative by suppressing formation of reactive oxygen species and preventing release of cytochrome c from mitochondria [41]. Furthermore, neuroprotective effects have been reported for quercetin (a phytoestrogen) and 17β-estradiol, which are probably not mediated via ERs but are rather based on their antioxidant and free radical scavenging properties [42].

Estrogenic activity of some compounds of the TG-decoction and their derivatives and/or metabolites may also indirectly contribute to the observed antiaging activities. Upon binding ERs, estrogen upregulates expression of antioxidant enzymes via intracellular signaling pathways [41,43]. Recently, ERs have been identified in mitochondria [44] and E_2 _treatment has been reported to increase the level of ERα in mitochondria and to modulate mitochondrial function, resulting in greater energy-producing capacity and decreased reactive oxygen species production [45].

With regard to TCM, TG-decoction regulates balances of yin-yang, exterior-interior, cold-hot and deficiency-excess, and thus ameliorates menopausal symptoms and overall body health, and contributes to general well being. To shed light on the mechanism of efficacy of this herb mixture in treating menopausal syndrome and in promoting global health, further studies are in progress, including those aiming to define the precise composition and identify targets of this herb mixture.

## Conclusions

TG-decoction balances female hormones, exhibits antiaging and antioxidative activities, and upregulates ERs. On the one hand, it shares some similar effects with conjugated estrogens. On the other hand, its mechanism of action seems to be more complex. Future studies should explore the constituents of TG-decoction and investigate their effects on expression of relevant genes and signaling pathways.

## Abbreviation list

E_2 _: estradiol; ERα: estrogen receptor alpha; ERβ: estrogen receptor beta; ERs: estrogen receptors; FSH: follicle-stimulating hormone; LH: luteinizing hormone; MDA: malondialdehyde; ROS: reactive oxygen species; SOD: superoxide dismutase; T-AOC: total antioxidant capacity; TCM: traditional Chinese medicine; TG-decoction: Tiáo Gēng Tāng

## Competing interests

The authors declare that they have no competing interests.

## Authors' contributions

LWX: principle investigator of the study, coordinating the study, analyzing the data and preparing the manuscript. LK: evaluating the data, correcting the manuscript. TTZ: coordinating the study and correcting the manuscript. SNL and YYM: Performing the study and analyzing the data. ZS, JM and XL: Performing the study. ZJS: Supervising the work and coordinating the study. All authors read and approved the final manuscript.

## Pre-publication history

The pre-publication history for this paper can be accessed here:

http://www.biomedcentral.com/1472-6882/11/137/prepub
